# Can a Learning Companion Be Used to Continue Teaching Programming to Children Even During the COVID-19 Pandemic?

**DOI:** 10.1109/ACCESS.2020.3020007

**Published:** 2020-08-27

**Authors:** José Miguel Ocaña, Elizabeth K. Morales-Urrutia, Diana Pérez-Marín, Celeste Pizarro

**Affiliations:** 1 Ejército Ecuatoriano Ambato 180156 Ecuador; 2 Universidad Técnica de Ambato 27905 Ambato 180103 Ecuador; 3 Ciencias de la Computación, Arquitectura de Computadores, Lenguajes y Sistemas Informáticos y Estadística e Investigación OperativaUniversidad Rey Juan Carlos 16776 28933 Madrid Spain; 4 Matemática Aplicada, Ciencia e Ingeniería de los Materiales y Tecnología ElectrónicaUniversidad Rey Juan Carlos 16776 28933 Madrid Spain

**Keywords:** Learning programming, programming environment, primary education, COVID-19

## Abstract

Learning how to program in Primary Education has attracted significant research in recent years. It is unclear though how programming environments and languages should be adapted to children to achieve better learning and use, but one trend seems to be the use of Scratch. The question in this paper is what programming environment can be used to continue teaching programming to children who have already been taught Scratch for years. This paper’s proposal is that students aged between 10 and 12 can benefit from interacting with a friendly learning companion using p-code such as Alcody. The hypothesis is that students (aged between 10 and 12) with a knowledge of Scratch will be able to significantly improve their scores by using a learning companion to teach them how to program even during the COVID-19 pandemic. To check the hypothesis, an experiment was carried out during the 2019/2020 academic year with 137 students in Ecuador. A significant improvement in the scores of the students was recorded together with high satisfaction.

## I. Introduction

Programming can be defined as a means of communicating with a machine using a language that it ‘understands’ to get the machine to do something to solve some problem [1]. However, some programming languages are very technical in their syntax and lack expressive power. This could be the reason why teaching programming is a complex task that should be researched [1]–[3].

The effort is worthwhile, as students become active builders of their own programs and it helps them improve their understanding of how the digital world in which they live works [4].

According to some researchers, learning how to program can enable students to develop higher cognitive skills [5]–[7] and they can even develop a new way of thinking called “computational thinking” (CT) [8]. CT allows the use of computer procedures to solve everyday problems. It is not necessary for students to want to become Computer Engineers but they can take advantage of thinking like a Computer Engineer in their lives. More importantly, it has been studied that the earlier children start learning how to program, the higher they can develop CT and other skills [9].

Given the difficulty of teaching programming, several approaches have been tried such as: (i) using a visual block-based environment with a multimedia language like Scratch [10], (ii) programming robots such as Lego Mindstorms [11], (iii) creating programs using textual programming [12], or (iv) using unplugged activities without using computing devices [13].

The focus of this paper is subsequent to these approaches having been tried. The proposal is to incorporate a learning companion [14] into a programming environment co-designed with children to keep learning how to program. The use of learning companions has benefited from significant research in recent decades. A learning companion can be defined as a computer agent in the educational environment that supports students. For this aim, the learning companion can learn from the student (“learning by teaching” paradigm, e.g. Betty’s Brain [15]), it can provide students that take the role of a teacher with suggestions, it can collaborate (sharing responsibility of the task with students) or compete against human students, it can just watch students’ actions so that students do not feel alone, or it can try to empathize with students so that students feel understood (e.g. Jake & Jane [16]).

In the literature review, no learning companion has been found that can be used to teach children how to program. Therefore, the design and development of a new educational environment to teach Primary Education children how to program with the Alcody learning companion was started [17].

Alcody is the result of a co-design with 66 children [18]. The interface for both the environment and the learning companion can be chosen by the children, as they will be the users. A motivating and pleasant environment is necessary to have a fruitful learning experience [19].

Moreover, Alcody follows Papert’s constructionism theory [12]. According to constructionism, the way to teach, in this case programming, is with an ‘object-to-think-with’. Alcody is based on the core idea that children need to think to be able to create programs with a certain goal [12], [19]. Children can input their programs using the Alcody p-code language. It compiles the program and provides immediate feedback.

In this case, the ‘object-to-think-with’ is the program. In the chat, Alcody can ask students something like “Write a program to show your name on screen.” Students must think and write the program with the input/output instructions. They are not going to be given the correct solution as proposed by constructionism; they must reach their own solution with the help of the Alcody’s tutorials and debugging possibilities.

Alcody also follows a gamification approach [20], [21] with medals as rewards and riddles as challenges. Students can have the emotional support of a learning companion with a robot form. Six emotions are supported by Alcody: happiness, sadness, fear, boredom, anger, and surprise [22]. The idea is that students can even use Alcody in online situations without face-to-face interaction.

The Research Question 1 and the hypothesis Ha are as follows:

RQ1 – Did students (aged between 10 and 12) increase their learning scores in a pre-post test using Alcody before and during the COVID-19 pandemic?

Ha – Students (aged between 10 and 12) with a knowledge of Scratch will be able to significantly improve their scores by using a learning companion to teach them how to program even during the COVID-19 pandemic.

Research Question 2 has a more descriptive nature and is formulated as:

RQ2 – How satisfied were students interacting with Alcody before and during the COVID-19 pandemic? What was their opinion of Alcody? Were they motivated to use Alcody even during the lockdown?

The results of an experiment with 137 students aged between 10 and 12 in Ecuador with a knowledge of Scratch support the hypothesis. A significant improvement in students’ scores is recorded together with high satisfaction even during the COVID-19 pandemic when they used Alcody to continue learning to program.

It is important to highlight that, when children were using Alcody, the teacher adopted an assistant role in case children did not understand something. Children were directly interacting with Alcody, which was in charge of asking the students to write the programs, debug any possible mistakes and provide help should they needed it.

The paper is organized into eight sections: Section II presents related work; Section III presents the theoretical framework; Section IV describes the Alcody environment and learning companion; Section V focuses on the experiment carried out; Section VI presents the results; Section VII presents the discussion and threats to validity; and, Section VIII ends the paper with the main conclusions and lines of future work.

## II. Related Work

### A. Teaching Programming

Pioneered by Papert [12], teaching programming to children had received a great deal of attention during the 1980s and beginning of the 1990s [19]. However, by the late 1990s and early 2000s interest diminished. Three reasons explain why: (i) complexity of teaching programming [1], (ii) lack of instructors and subject-matter integration [23], and (iii) the creation of new multimedia interfaces that moved teaching away from constructing programs to teach how to use the visual interfaces of the programs as users [24].

In the last decade, a renew interest in teaching programming to children as a means of expression has arisen [25]. However, until now, there has been little evidence on how to design a successful coding experience for children based on their needs, age and attitude [26].

Given the large volume of approaches and the vast possibilities for each [27], the focus is on the most appropriate learning activities for children to achieve the most intuitive and engaging experiences [26] as well as being more effective (higher learning gains).

**FIGURE 1.**
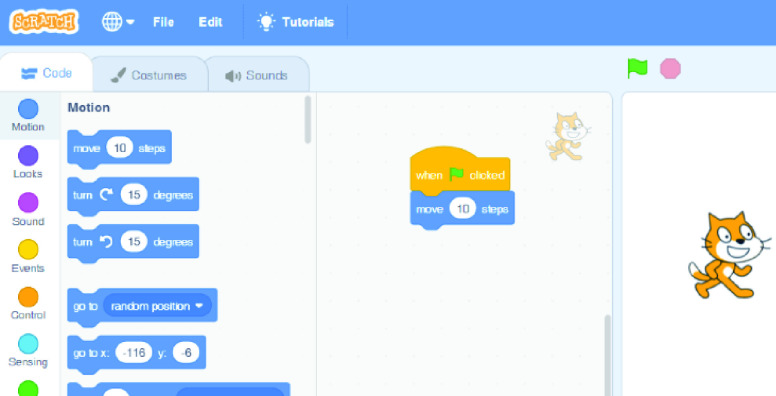
Scratch user interface^1^.

The constructionism approach [12], [19], [28] seems to provide a valuable learning experience of coding that increases the interest in coding as well as an understanding of the fundamental concepts of problem-solving [29]–[31]. Children’s learning experiences in constructionism-based coding activities seem to be positive [19]. They used Scratch to allow children to construct a game as a socially meaningful artifact. Moreover, for the construction of the artifact, they argued that children had the opportunity to plan, think, debug, collaborate, communicate and reflect on their experience. Figure 1 shows a snapshot of the Scratch programming environment and language [10].^1^https://scratch.mit.edu

Scratch is grounded in both construction-based activities with the use of blocks and gamification with the design of multimedia games. This could explain the good results achieved in children learning to program for the first time in this environment [32]. Children using Scratch usually exhibit highly positive attitudes and motivation [19].

Instructions in Scratch are grouped into several categories: motion, looks, sound, events, control, sensing, operators, variables and others. Each category has a different representative color.

Each instruction is contained in a block with the parameters in editable spaces. Children choose one block with the instruction and drag it to the program editor. The blocks fit together like pieces of a puzzle. The program is run by double-clicking on the program (i.e. the set of blocks) or clicking the green flag and the result is displayed on the screen. For instance, the program’s goal may be to move the cat 10 steps as in Figure 1, so when the child clicks the green flag, the cat moves 10 steps.

Scratch is a highly visual environment oriented toward multimedia with many possibilities such as changing backgrounds, configuring the characters or adding sounds. In this environment, it is impossible for children to make errors in syntax as they do not write instructions, they just enter the value of the parameters in the indicated spaces. Possible errors result from an incorrect choice or order of the instructions. Other than watching the value of the variables, there are no integrated debugging possibilities such as breakpoints, or an explanation of the result if it is not as expected.

### B. Learning Companions

A Pedagogic Conversational Agent (PCA) can be defined as a representation or personification of the teacher or the student in a computer that guides users through multimedia learning environments [33], [34]. Its main goal is to engage, motivate, and guide learners through the learning process. In this context, an agent is understood as a simulated character in the computer that can exhibit human behaviors. The virtual character normally takes the form of a human, animal or robot and interact with voice, text, gestures or a combination of all three.

The use of PCAs can increase learners’ motivation, engagement and self-efficacy [35]. Some other benefits reported as a result of using PCAs in education are: the Persona effect [36], just the presence of the PCA in the educational environment can have a positive effect on students’ perception of the learning experience; the Proteus effect [37], students can have higher motivation if they want to resemble the PCA of the educational environment; and, the Protégé effect [38], students can make a greater effort to teach their PCA than to learn from their PCA.

There are many different PCAs applied to different domains and to develop competences from Pre-Primary to Higher Education [39], [40]. Some domains in which PCAs have been used successfully are: Science [36], Math [41], Languages [42], Storytelling [43], Social skills [44], Operating Systems [45] and Programming [46] for Higher Education in object-oriented languages such as C++ [47] and JAVA [48], [49], procedural languages such as C [50], database management such as SQL [51] and the tangible programming Tern in Museums [52], [53].

Different agents can be introduced to support scaffolding activities in learning programming languages and problem solving such as teachers to support learning or harvesting agents to collect resources [48].

However, to the best of our knowledge, no PCA had been created to teach programming to Primary Education children until I-Prog [17]. One of the main characteristics of pedagogical agents is interactivity [54]. They not only provide answers and additional learning material, but also generate questions and propose solutions. I-Prog was created with the core idea of interacting with students following the MECOPROG methodology [55] to teach them basic programming concepts by solving problems and making them think.

Regarding the impact of using PCAs on improving students’ learning gains, mixed results have been published [56]. Some authors have reported that using an agent increased students’ scores [45], whereas other authors have reported that using an agent did not have any effect on students’ scores [34].

On the other hand, the impact of using PCAs on improving students’ satisfaction and motivation is proved [52], [57]. Given the importance of social and emotional aspects in e-learning [58], [59], the evolution of PCAs as learning companions was contemplated. Some authors have considered that PCAs were unable to regulate emotional responses, unlike learning companions that are specifically designed to support the student not only as interactive lifelike characters that guide them but as emotional “friends” [60], [61].

Learning companions can also be considered as a subtype of PCAs in which the emphasis is on the companion role of the agent, in an individual or collaborative use [62] in contrast to agents in which the main role is a teacher or even a student role [39]. Studies have shown that conversational agents acting as educational companions or tutors can facilitate learning [63], [64].

A learning companion can be defined as a “robot or a virtual conversational agent that possesses a certain level of intelligence and autonomy, as well as social skills that allow it to establish and maintain long-term relationships with users” [65]. Children have sufficient imagination to transform reality into fantasy situations, to give personality and life to inanimate characters on the screen, conjure up imaginary companions, invisible friends and interact with them [66].

The use of a companion could improve the interaction strategy between children and the application [60]. It is also important to design the companion (in general, agent) to be attractive to students, as if students dislike the agent’s visual appearance, it could have negative effects on the learning gains [34].

Many European projects are devoted to studying the effects of using companions in computer-based education such as the LIREC^2^ and EMOTE^3^ projects. The literature offers many samples of companions and good results in their interaction with children such as Huggable, a teddy bear fantasy for pediatric care [67] or Emily, a companion for dialog with children [68]. Both are based on enjoyable interaction using three factors to engage children: personalization, adaptation to the children’s skills (interaction should be challenging but not overpowering) and supporting social interaction (empathetic personality and social skills seem particularly relevant).^2^http://lirec.eu/project^3^http://www.emote-project.eu/

There are three main strategies that companions can adopt when interacting with children in educational environments [60]: a sympathetic strategy to guide them to a positive state, a cheerful strategy to celebrate all their progress and an inquisitive strategy to find out the cause of their emotional state.

In regard to student-companion dialog—the Conversational Interaction Strategy, CIS—it is also important to highlight that there are several phases that need to be considered: the start and end of the dialog, turn management (taking turns to listen and speak), active waiting, and interruptions [69].

CIS is designed to resolve failures in the dialog system and deal with problematic conversational features such as listening after ceding a turn or being polite when interrupted. The following strategies can be adopted in student-companion interactions [60]: being surprised if the user gives new information, confused if they give contradictory information, listening to whether they are talking just to interject, if they are idle or standing back without answering, or interrupted if the dialog stops and the turn is taken.

**FIGURE 2.**
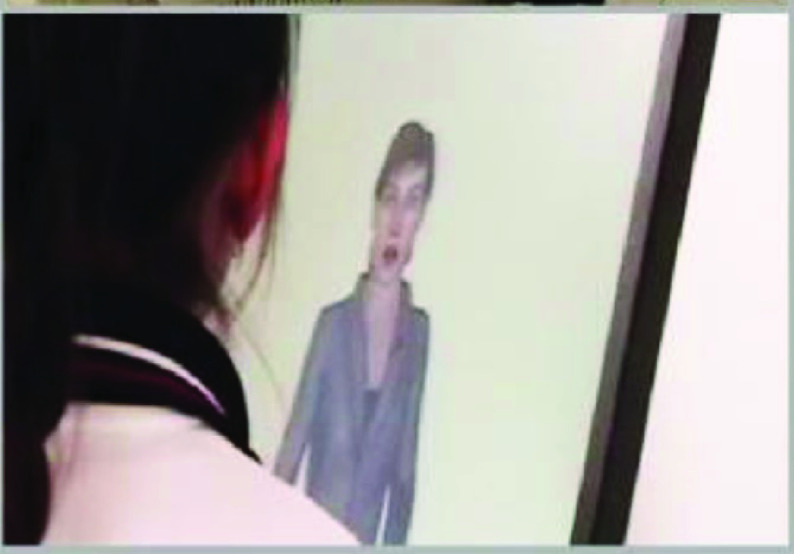
Sample learning companion (source: [60]).

Figure 2 shows a sample companion playing with a child in a collaborative co-located learning game.

As can be seen, the companion adopts a human form and talks directly to the student. The expectations in this case are very high as the situation is realistic. It is not advisable, however, to offer such realistic companions if the quality of the dialog between students and the companion is not of a sufficient standard. If the standard is not sufficient, animals or robot forms can be used [60].

Finally, it is important to highlight the study completed with Emmy, a learning companion to teach interpersonal safety to children. In a test following the use of Emmy for six months, children were able to correctly choose the safe response option. Moreover, parents reported that their children used more safety strategies after using Emmy [70].

### C. Gamification

Another major factor to consider in achieving an intuitive and engaging experience for children learning how to program is gamification. Children should learn how to code in a fun and playful manner [71]. Gamification is a tool that consists of using game design elements in non-gaming environments to increase motivation in tasks [72], [73].

Gamification makes activities more fun and engaging [74], [75]. Gamification in education is a growing topic attracting a great deal of research in recent decades because of its potential not only in increasing students’ motivation but also their performance [76], [77].

Researchers have found a correlation between higher performance and applying gamification techniques to certain activities, although it needs to be accompanied by a successful game design and not only isolated achievements [78].

Gamification in education can be successfully applied to all levels from Primary Education [79], High School [80], to college [81] and in diverse fields of knowledge such as Biology [82], Programming [83], numerical methods [81] or electromagnetism [84].

Although there is no closed list of elements for gamification, the most commonly used include [85], [86]: a) Rewards with points, stars or badges; b) Indicators of progress with levels passed, progress bars, leaderboards or medal displays; and, c) Meaningful and fulfilling challenges. For example, the gamified Microsoft help, Microsoft Ribbon Hero [87], uses points, badges and levels to motivate people to explore Microsoft Office tools. Jigsaw [88] teaches Photoshop by using jigsaw challenges.

Using gamification to teach programming has had good results by using rewards such as points and badges [89]. Making a game offered higher motivation, enthusiasm, and engagement to children who overcame difficulties with learning how to program such as boredom or confusion [90]. A gamified approach with positive results to teach algorithms, control structures and variables to children is presented in [91]. The effectiveness of learning theories using a gamified Android app in teaching C programming is also reported by [92]. A more complete review study on using gamification to teach programming can be found in [93].

## III. Theoretical Framework

**FIGURE 3.**
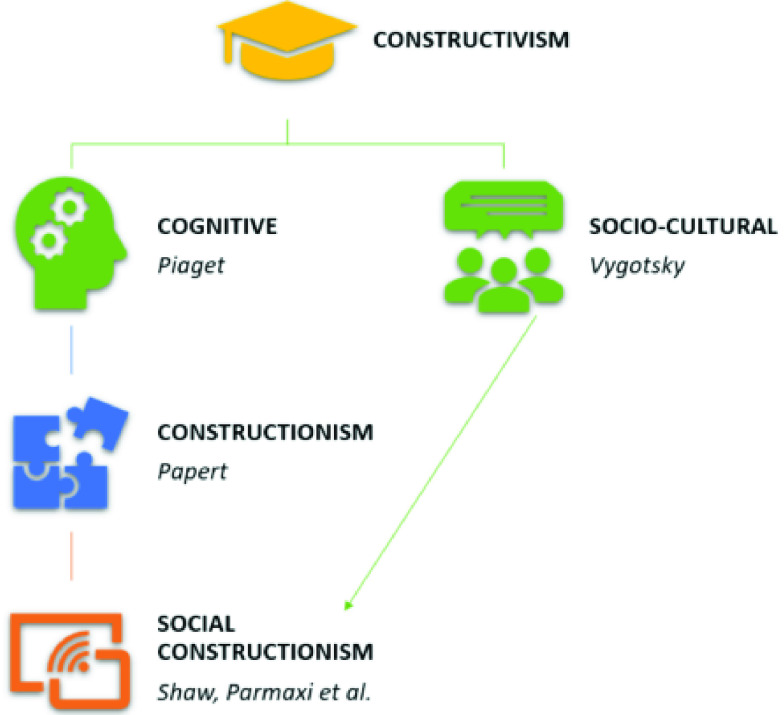
Background for the theoretical framework of teaching programming with learning companions.

Constructivism (see Figure 3) is a paradigm that comprises several learning theories that have the core idea of students constructing their own learning in common [94]–[96]. Among the theories, two can be highlighted as having a theoretical background: the individual cognitive aspect of constructivism of Piaget’s theory [97] and the socio-cultural aspect of constructivism of Vygotsky’s theory [98].

Piaget provides a theory to understand how children think and do things according to their developmental level. Learning should be considered as an individual process that occurs in the mind of each person.

To Piaget, knowledge is not data to be provided to the child to memorize, it is seen as an experience that each child acquires through interaction with the world, people and things [99]. This is why, when teaching to children, it is important to consider their previous knowledge and their individual preferences and features and not provide just data but to ask the child to experience the learning.

This individual approach, however, overlooks important aspects when teaching programing to children as it does not consider either the social component of interacting with others or the context and media.

Vygostky [98] provides the social aspect to constructivism. He stated that knowledge is co-constructed and that individuals learn from one another. The environment should be adapted as it has a key influence on learning.

Papert [12] follows the cognitive constructivism of Piaget as he also considers learning to not be a mere transfer of information from a sender to a receiver. Papert extends Piaget’s theory by taking the context and media into account. For Papert, learning is more effective if a concrete entity is built (“an object to think with”) and this is the core idea of constructionism.

In Papert’s own words, constructionism can be defined as “Constructionism—the N word as opposed to the V word— shares contructivism’s connotation of learning as ‘building knowledge structures’ irrespective of the circumstances of learning. It then adds the idea that this happens especially felicitously in a context where the learner is consciously engaged in constructing a public entity, whether it’s a sand castle on the beach or a theory of the universe” [28].

Papert’s constructionism is more pragmatic than Piaget’s cognitive constructivism or Vygostky’s socioconstructivism [100]. According to constructionism, students will not learn programming just by listening to their teacher or reading a book, they need to program. Through coding, using computers, children have an ’object-to-think-with’ and thus, to learn [12]. In the LOGO environment, programming is introduced when children interact with the Turtle, which acts in response to the children’s commands.

According to Papert’s constructionism (based on Piaget’s cognitive constructivism), personalization and adaptation options are necessary for any teaching environment. The idea is not that students learn a correct solution, with all of them following the same steps and procedures, but that each student reach a different optimum solution according to their thought processes, which is the correct way to learn [101].

This is relevant for teaching programming, as students should not be given “a correct solution”. There is no a correct solution. Students should be guided as needed to reach their own correct solution [1].

Natural language interaction can be used as a way to reach that individual correct solution [102]. As Papert said: “We learn better by doing…but we learn better still if we combine doing with talking and thinking about what we have done.” “Life is not about ‘having the right answer’ – it is about getting things to work” [103].

Social constructionism is an evolution of constructionism with an emphasis on the social setting whilst engaging the student in the artifact construction [104], [105]. Almost three decades after Papert’s formulation of constructionism, his ideas remain relevant with coding being one of the areas in which (social) constructionism has been mostly applied [106], [107] in both formal and informal educational contexts [108].

By creating social meaningful artifacts such as games, children seem able to learn how to program as described in Section II.A. Using computers to teach programming to children, constructionism theory can be easily put into practice [109].

Social constructionism is also valuable to enhance the social setting [110]. Children can become producers of their programs in a social context by interacting with other children. Even where face-to-face interaction with children is not possible, interacting with a learning companion (as described in Section II.B) can enhance the experience of coding and creating programs.

A final aspect of constructionism in relation to feelings and emotions, learning can also be seen as the expression of personal feelings [100]. There is a relationship between what the child feels and their ability to code. Therefore, emotions should be taken into account to improve how to teach programming [19].

Highly motivated children can handle the cognitive load better and construct their artifacts better. In the words of Papert, “You can’t learn bread-and-butter (basic) skills if you come to them with fear and the anticipation of hating them” [12]. A learning companion should be able to support and manage students’ emotions to provide a pleasant and motivating environment that leads the learning experience to success.

## IV. Alcody

To the best of our knowledge, no previous use of learning companions to teach programming to children has been published. The previous good results of companions in other teaching situations [63], [64], [67], [68] encourage exploring the possibility of using companions to teach programming to children.

This could be particularly useful after they have started learning programming with a program such as Scratch [10]. As described in Section II, Scratch is grounded in constructionism [12], [19], [28] and gamification [71] approaches. It is a very powerful tool in creating engaging and effective learning experiences for children.

However, after being taught with Scratch for several years, motivation may decrease and a more in-depth learning of programming concepts should be examined. This is the reasoning behind proposing the use of a learning companion, based on a similar constructionism and gamification to Scratch but with natural language interaction rather than drag and drop to build the programs.

**FIGURE 4.**
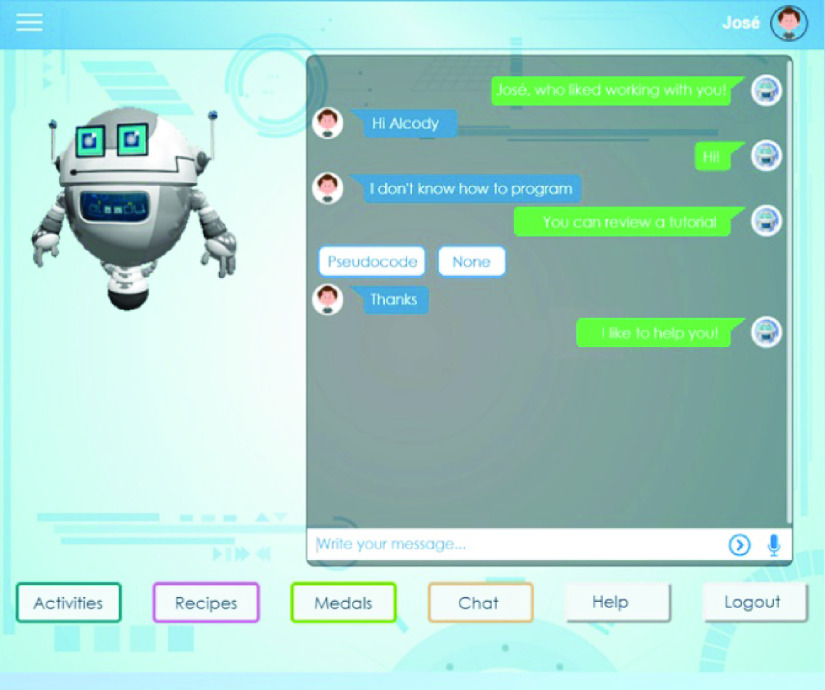
Alcody’s user interface^4^.

The companion called Alcody is based on constructionism because it asks students to build a program (see Figure 4). The difference is that students have to type p-code into the program [111]. This is thought to be suitable for children aged over 10 with prior knowledge of Scratch.^4^https:/alcody.site

Alcody is available online, in Spanish, at alcody.site (a demo can be accessed with the username “diana” and the password “123”). All images in the paper have been translated into English for non-Spanish speaking readers.

Following the MEDIE methodology [40], a co-design with 66 children aged between 10 and 12 took place to develop the first prototypes of Alcody [18]. Children were asked about the form of the learning companion (e.g. robot, animal, boy, girl, etc.), the colors and the interface possibilities.

Children can choose between six different futuristic robot forms for the learning companion with 16 different pastel colors for the environment (light and dark blue, purple, pink, yellow, orange, green, grey and white) as these factors were important for them to like the environment and learning companion more [18].

The learning companion has anthropomorphic features with a head, body and limbs. Its mood is calm, friendly and empathetic. It seeks to help children to program through its presence and messages by mainly following a sympathetic strategy to guide users to a positive state and a Conversational Interaction Strategy focused on taking turns.

The possibilities of the educational interface are: to make *activities*, to follow *recipes*, to see your *medals*, to *chat* to solve exercises, *help* with six tutorials to teach children p-code and how to use Alcody and *logout* to close the session and save the progress.

**FIGURE 5.**
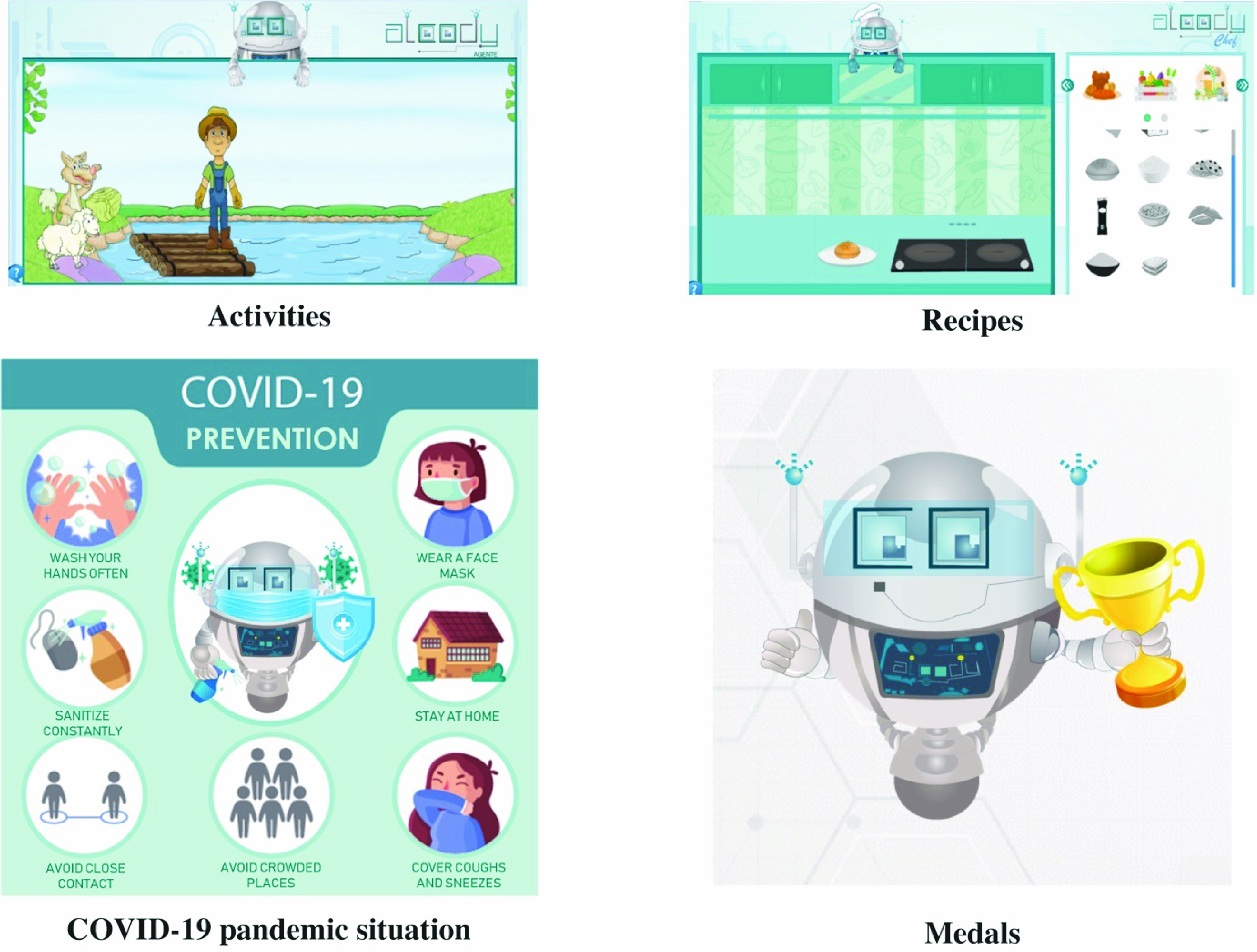
Alcody’s activities and rewards.

The *activities* are designed to give children some play time given the proven benefits of gamification [71]. Three games are currently included in Alcody (see Figure 5):

• The riddle of wolf, sheep and cabbage: a farmer must move to the other side of the river with the wolf, sheep and cabbage. In each trip, users can only take the farmer and the wolf, sheep or cabbage, and must take into account that if the sheep is left alone with the cabbage, it will be eaten and if the sheep and the wolf are left together, the wolf will eat the sheep.
• Jumping frogs: children have to click on the frogs to move them to the next stone. Frogs can only pass over another frog once. In order to win, the three brown frogs should be moved to the right of the pond and the green frogs to the left.
• Missionaries and cannibals: on one of the riverbanks, there are three missionaries and three cannibals. The aim is to transport the six people to the other riverbank using the boat. The boat is only capable of transporting up to two people at a time. There cannot be more cannibals than missionaries, and the boat cannot be moved without anyone moving it.

The option to follow *recipes* is based on the so-called MECOPROG methodology [55]. According to it, programming is like cooking, and a program is like a recipe. This is apt because, when programming, the computer or robot must follow the instructions one by one to execute the program (a sequence of instructions with data). In the case of cooking, the chef must follow the lines of the recipe (like the instructions of a program) one by one to produce a meal (like the result of the program).

The program’s variables are like the recipe’s ingredients. The computer’s memory is like the pantry in the kitchen. The core idea is that children at those ages should enjoy cooking and understand the process of cooking.

Several recipes are proposed in Alcody such as cooking pasta, an omelet or a hamburger (see Figure 5). Each time students complete one of the activities or a recipe, they are given a medal or trophy. Rewards are very important in gamification to increase students’ motivation.

Given the current COVID-19 pandemic and the educational nature of Alcody, the importance of adding a new *prevention* activity has been considered. Alcody tells children to wash their hands, clean everything, stay more than two meters away from other people, use face masks, stay at home as much time as possible, avoid crowded places, and cover their mouth with their elbow when coughing or sneezing.

**FIGURE 6.**
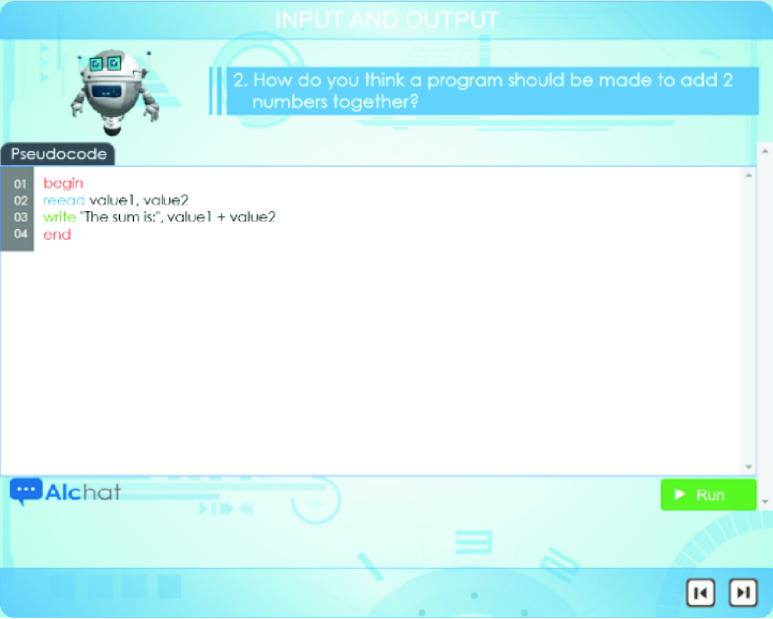
Sample Alcody p-code program.

Regarding the *chat*, Alcody always starts the conversation by asking students to solve a problem through programming. For instance, as shown in Figure 6, Alcody is asking students to write a program that allows two numbers to be added together. Students must write the program in p-code in the text area provided and press “Run”.

The core idea comes from Papert [12] as it is believed that students must think in order to program. They should think of the need for variables, how many, which instruction types are needed, and their sequence. They must also think of the p-code and write it to check whether their program produces the expected result.

Alcody compiles the program. It is not interpreted line by line so students have to think the program as a whole. When students finish writing the program, they have to press the “Run” button, and Alcody produces the result. If the result is as expected, Alcody congratulates students and asks them for another program that covers the programming curriculum concepts given by their teachers.

**FIGURE 7.**
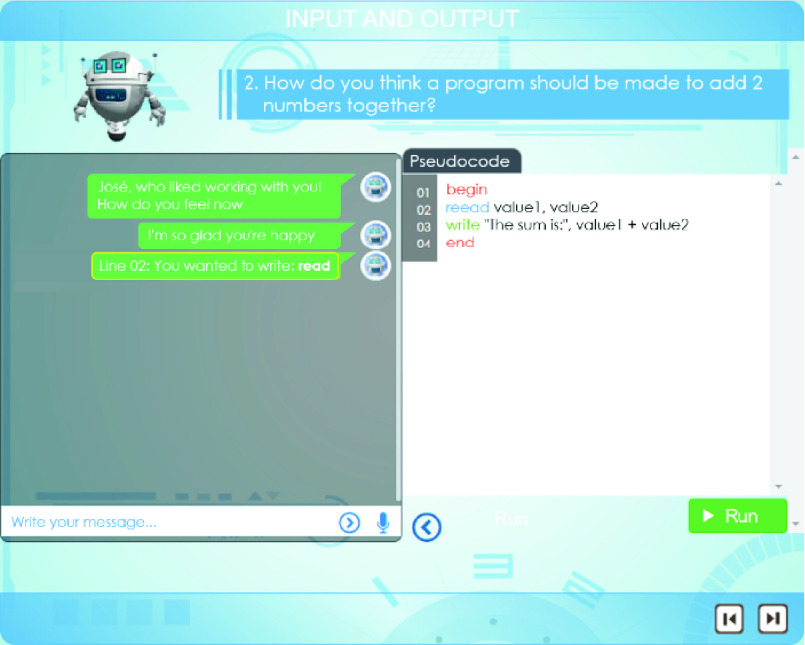
Sample help provided by Alcody’s debugging possibilities.

Teachers are always in charge of creating, modifying or removing exercises in Alcody’s database of exercises. Teachers usually provide between three and six exercises per programming concept. The idea is that students can practice the programing concepts with real programs that they type in p-code with immediate feedback and debugging possibilities. If the expected result of the program is not produced, Alcody’s debugger can help students, as shown in Figure 7, by talking to students to make them aware of their mistake, without judging them: this is informative feedback to make students think again and modify their program until the expected result is achieved.

If students have problems with the p-code syntax or anything related to Alcody, they can press the “Help” button to access one of the video-tutorials created to help them.

Table 1 summarizes Alcody’s simplified p-code instructions.

**TABLE 1:** Alcody p-Code

Instruction	Explanation
begin	It marks the beginning of a program
end	It marks the end of a program
write “t” $\vert $ var	To show something on screen (text between “ “ or the data from the name of the variables)
read var	To read a value from the keyboard and save it in variable var
if cond then $X$ else $Y$	It marks the beginning of a condition with two branches (X,Y) depending on cond

Alcody provides children with personalization and automatic emotion management possibilities [22]. Children can modify various aspects of their profile such as the name Alcody will use in text and audio messages, their profile picture; the platform’s base color from a choice of 16 for all the platform’s elements, and one of six different configurations of color and shape.

**FIGURE 8.**
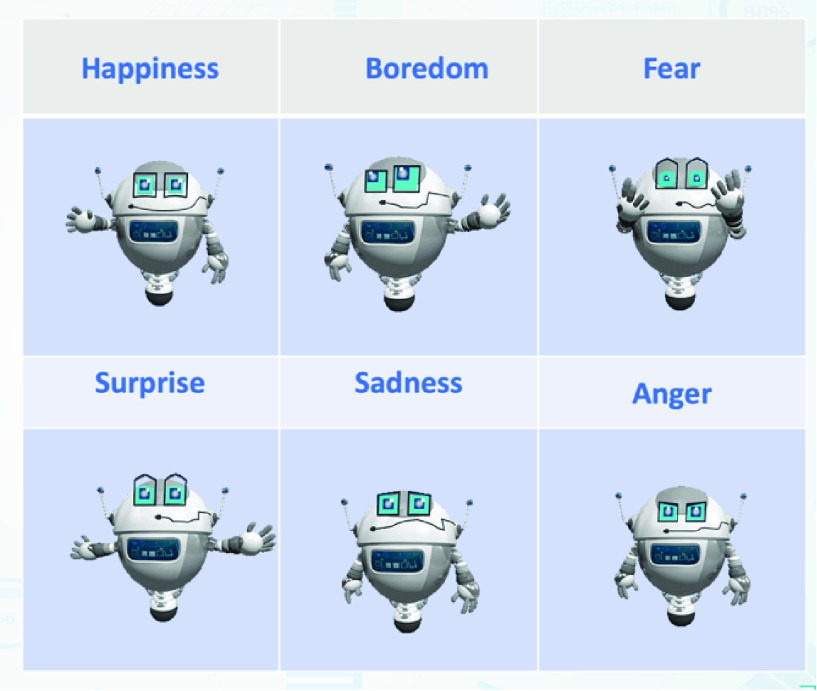
Alcody’s emotions.

Finally, as shown in Figure 8, six emotions are considered by Alcody: happiness, boredom, fear, surprise, sadness and anger to try to empathize with student.

Children type in the chat and Alcody identifies the words written to interpret students’ emotions and responds with a message based on the emotion detected combining a sympathetic and cheerful strategy and a listening Conversational Interaction Strategy [60]. Alcody displays empathy with the emotion detected in the message written in the chat through its facial and body expressions [22].

**FIGURE 9.**
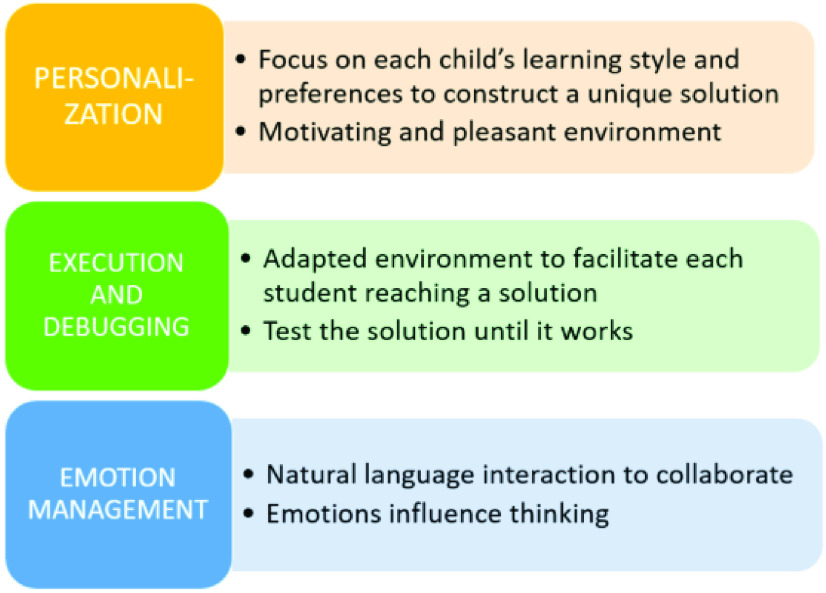
Connection between Alcody and constructionism.

Alcody and their learning materials have been designed according to the theoretical Papert constructionism theory described in Section III. The core idea is that learning is achieved through taking both the use of computers and the interaction with the learning companion (context and social aspects) into account. Figure 9 illustrates the relationship between the three main factors of Alcody and Papert’s constructionism.

Personalization is grounded both in the individual view of Piaget that each child should think, according to their development stage, to reach to a solution with their own mind. However, not everything is limited to the student’s mind. Context is important for Alcody, as it is the means to help the student think by following Papert’s constructionism. With Alcody, children can learn by constructing their own unique artifact, in this case, their program solution.

A co-design effort has been made in Alcody so that children have had the opportunity to design Alcody according to their preferences and needs to provide a motivating and pleasant environment for a fruitful learning experience [19].

Execution is grounded in the idea that, if students interact in natural language with the companion, it can guide them to their “correct solution”. No correct solution is provided by Alcody as no correct solution exists for constructionism. Children are helped to reach a correct solution. They can execute their programs (object-to-think), “following true constructionism they are trained to master the process of searching for errors and correcting them.” [1].

The goal as Papert [12], [103] said is that although the first time they try their solution it does not work, they keep thinking about how to solve errors and trying again. In this, they are helped by Alcody’s debugging possibilities and are encouraged to act intentionally to improve the code as Papert claimed.

Finally, regarding emotion management, Alcody dialogs with the students to help them reach their optimum mental state. The core idea is the same as Papert’s constructionism as if students are sad or frustrated, they are not focused on creating the solution as emotions influence students’ thinking [12], [19].

## V. Methods

The study is a pre-post test single group design. Following an adaptation of the guidelines to report experiments in Software Engineering written by [112], [113] this section is structured as follows: A. Goal, B. Participants and Context, C. Experimental Material, D. Tasks, E. Variables, F. Hypothesis, G. Procedure and Tools, H. Analysis Procedure, and I. Validity and Reliability.

### A. Goal

The main goal of the study was to find out whether students with prior Scratch knowledge would keep learning programming even during the COVID-19 pandemic with the help of the Alcody learning companion. The learning scores of the students served as a quantitative measure to test whether their learning with Alcody had been effective before and during the COVID-19. The Research Question 1 (RQ1) was as follows:

RQ1. Did students (aged between 10 and 12) increase their learning scores in a pre-post test using Alcody before and during the COVID-19 pandemic?

This quantitative approach was combined with a qualitative approach taking into account the motivation, satisfaction and general opinion of the students when using Alcody before and during the COVID-19. The descriptive Research Question 2 (RQ2) was as follows:

RQ2 – How satisfied were students interacting with Alcody before and during the COVID-19 pandemic? What was their opinion of Alcody? Were they motivated to use Alcody even during the lockdown?

### B. Participants and Context

The experience was conducted in the 2019/2020 academic year with 137 students (aged between 10 and 12) in a Primary Education school in Ecuador. Almost half the students were girls.

The school was chosen for two main reasons: (i) the Head of the school was willing to authorize the experiment provided that all children used the learning companion to learn programming, and (ii) although not compulsory in Ecuador, children in this school learn programming using Scratch from the fourth year of Primary Education (8-9 years old).

During the period before COVID-19, students were in class (four groups of 32, 36, 34 and 35 students) with their teacher and two researchers who were responsible for conducting the experiment. They were also the observers of the learning experience for the qualitative approach. The teacher was only to provide support if students had any issue. The teacher did not participate in the observation. Each child had one computer to use in the class.

During the lockdown, students were at their home. They connected online, in their group, with their teacher from their personal computer. All children had a computer, a similar digital skill using it, and an Internet connection at home.

Neither before nor during COVID-19 were children paid for using Alcody or given educational credits or higher scores by their teacher as a reward. They were committed to their classes as using Alcody was integrated into their lesson activities. The main incentive provided was the gamification approach of Alcody as described in Section IV.

No permission was granted by the school to record the sessions. The consent was to allow children to use Alcody and observers take notes. Confidentiality was assured, as children did not give their name to Alcody at any time. However, a code was assigned to them. They were told that it was very important that they remembered their code as Alcody asked for it as their username for all sessions. To ensure honest answers to questionnaires, that code was not requested. Children were reassured that the teacher would not penalize anyone because of their answers.

### C. Experimental Material

All educational materials were provided in the Alcody educational environment as described in Section IV. Children were not given additional material in class before COVID-19, nor during the lockdown.

Alcody’s curriculum covers knowledge about sequencing, memory, variables, inputs, outputs, and condition concepts. The goal is to teach students their meaning in programming, and to develop their skills to write programs using those concepts and their instructions in p-code.

Alcody teaches programming concepts in an interactive manner. Alcody will firstly ask students to write a program to say “Hello” on the screen. Not being focused on the theory about what a program is, the concepts are covered according to constructionism’s core idea of “learning by doing”. When the student writes a program that shows “Hello” on the screen, it demonstrates that they understand not only the program concept but also input/output. Children must therefore understand what a program is to create syntactically correct programs.

### D. Tasks

There was one one-hour lesson with Alcody per week. Before the lockdown, they were asked to work with Alcody during that hour in their group. During the lockdown, the class was at the same time and for the same length as before and with the same teacher connected online using a videoconference system.

Children were given the possibility of reading a tutorial if they did not understand the explanation provided by Alcody, as if they were in the classroom.

During the first two months, students used the activities or recipes for half an hour and read Alcody’s tutorials for the remainder of the class. For the remaining months, students programmed with Alcody on the concepts being studied for fifty minutes. As a reward, and to offer reinforcement, for the final ten minutes, students were given the option of either completing some activities or following some recipes. Table 2 shows the timing with the teaching activities asked of the children.

**TABLE 2:** Timing of the Teaching With Alcody

Period	Concepts covered with Alcody
September-October 2019	Pre-test, sequences with activities. Tutorials about Alcody’s p-code
November-December 2019	Sequences, memory, variables with recipes (activities to reinforce)
January-February 2020	Sequences, memory, variables and inputs/outputs with chat (activities and recipes to reinforce)
March-May 2020	Sequences, memory, variables, inputs/outputs and conditions with chat (activities and recipes to reinforce)

### E. Variables

The dependent variables of the pre-post test single group experiment are all related to learning programming concepts, measured by scores obtained by the students in tests in different months throughout the academic year. These scores (NotePre, NotePost1, NotePost2, Notepre_cond, Notepost_cond) were gathered with the tests described in Section V.G.

There was only one independent variable in the experiment, defined in a general way as exposure to the use of Alcody. This variable has different levels depending on the use of the learning companion until a certain month: i.e., September, February and June. More details in [114]. Table 3 contains the list of dependent and independent variables.

**TABLE 3:** Summary of Variables, Type (DV- Dependent Variable, IV – Independent Variable), Names and Description

Aspect	Type	Variable	Name
Learning of programming concepts	DV	Scores of test carried out in September	NotePre
	DV	Scores of test carried out in February	NotePost1
	DV	Scores of test carried out in June	NotePost2
	DV	Scores of condition questions on test carried out in September	Notepre_cond
	DV	Scores of condition questions on test carried out in June	Notepost_cond
Learning companion	IV	Use of Alcody	$\alpha $

### F. Hypothesis

In this section, first, a major research hypothesis is presented to later describe each of the working statistical hypotheses used to answer them.

Ha: Students (aged between 10 and 12) with a knowledge of Scratch will be able to significantly improve their scores by using a learning companion to teach them how to program even during the COVID-19 pandemic.

This improvement in their learning is measured in terms of the scores of the tests described in Section V.G. So, in this case, two different aspects are involved in this research hypothesis:

• First, as a general hypothesis, the interest is focused on the possible differences that may exist between the scores of the three tests performed in September, February and June. So, null and alternative hypotheses are as follows:
  \begin{align*} \left.{ {\begin{array}{l} H_{0af}:\mu _{i}=\mu _{j}=\mu \quad \forall i\ne j \\ H_{1af}:\exists \mu _{i}\ne \mu _{j} \\ \end{array}} }\right]\end{align*}
  Namely, the null hypothesis $H_{0af}$ is that there is no difference in the mean of the test scores, where $\mu _{i}$ is the mean of the score in the test at time $i =$ {September, February, June}. The alternative hypothesis $H_{1af}$ is that there are some differences in the means of the test scores. This hypothesis is solved by an ANOVA analysis, as described in Section V.H.
• Second, if a difference in the mean of the test scores is found, a second part takes place to understand exactly in which pairs of means in the scores those differences are:
  \begin{align*} \left.{ {\begin{array}{l} H_{0as}:\mu _{i}=\mu _{j} \\ H_{1as}:\mu _{i}\ne \mu _{j} \\ \end{array}} }\right]\quad \forall i\ne j\end{align*}

These are, in total, three different hypotheses contrasts as a post-hoc study (in these studies, in general, the amount is $\begin{aligned} \left ({{\begin{array}{cccccccccccccccccccc} n\\ 2\\ \end{array}} }\right) \end{aligned}$, with $n =$ number of levels of the factor). The null hypothesis $H_{0as}$ is that there is no difference between the mean of the test scores carried out in month $i$ and in month $j$. The alternative hypothesis $H_{1as}$ is that there is. These hypotheses are solved with Tukey’s HSD (honestly significant difference) test, as described in Section VI.B.

• In the case of the Condition concepts, two tests were carried out, in September and June so the null and alternative hypotheses are:
  \begin{align*} \left.{ {\begin{array}{l} H_{0ac}:\mu _{s}=\mu _{j} \\ H_{1ac}:\mu _{s}\ne \mu _{j} \\ \end{array}} }\right]\end{align*}
  where the null hypothesis $H_{0ac}$ is that there is no difference in the mean of the test scores in September and June, (namely $\mu _{s}$ and $\mu _{j}$). The alternative hypothesis $H_{1ac}$ is that there are some differences in the means of these test scores. This hypothesis is solved by a paired t-test analysis, as described in Section V.H.

It is important to note that differences in the contrast hypotheses are at a 95% confidence level.

### G. Procedure and Tools

A pre-post test procedure was followed for ten months. A condition of the school was that all students must use Alcody. The progress of the students’ programming knowledge was measured by asking all students to complete a pre-test at the beginning in September 2019 before using Alcody, a first post-test just before the lockdown in March 2020, and a second post-test in June 2020 during the lockdown.

All tests use the following questions focused on evaluating the students’ programming skills using the concepts covered in Alcody:

Q1. Write a program to display “Hello” on the screen. *(sequence, output concepts)*

Q2. Write a program to add two numbers. (sequence, memory, variable, input, output concepts).

Q3. Write a program that calculates the perimeter of a square from the user-input value of one side. *(sequence, memory, variable, input, output concepts).*

The pre-test (September 2019) and second post-test (June 2020) also included a question focused on assessing the use of conditions (it was excluded from the February 2020 post-test because the condition concept had not yet been taught):

Q4. Write a program that shows the highest number from two numbers given. *(sequence, memory, variable, input, output, condition concepts).*

**FIGURE 10.**
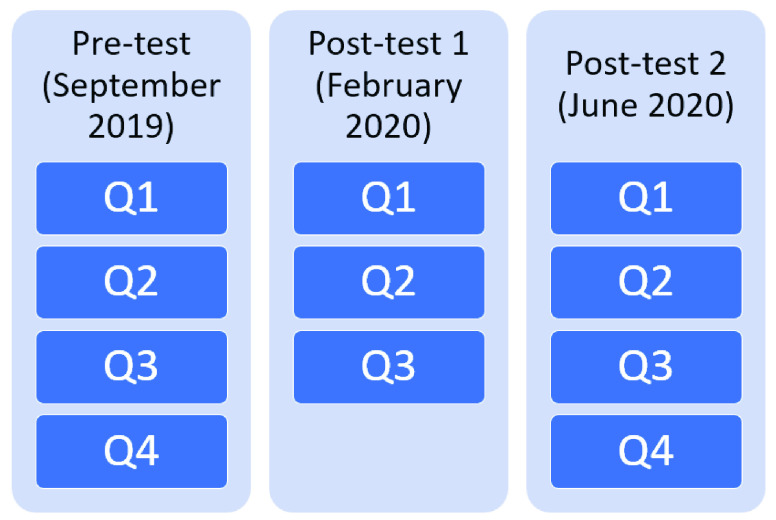
Questions analyzed in each test.

Figure 10 shows the distribution of questions in each test. Each question was evaluated from 0 (minimum score) to 1 (maximum score) following the rubric shown in Table 4. The minimum score for the test was 0 (all answers incorrect) and the maximum was 10 (all answers correct). The test score was the result of the weighted mean reflected in *Equation 1* as all questions were weighted the same in the test’s score. A pass mark was five.

**TABLE 4:** Rubric for the Test

Points	Assessment
0	Unanswered or incorrect
0.25	The start and/or end instructions are correctly used but the rest is incorrect
0.5	The start and end instructions are correctly used as well as the general sequence of instructions although the use of variables or syntax is incorrect
0.75	The start, end and sequence of instructions are correct as well as the use of variables with possible minor mistakes in syntax
1	The start, end and sequence of instructions are correct as well as the use of variables and syntax

Equation 1.

\begin{equation*} Note=\frac {10}{n}\sum \limits _{i=1}^{n} s_{i}\end{equation*}

where $n$ is the number of questions in the test and $s_{i}$ is the score for question $i$ of the test.

After the February post-test, all students were asked to fill in a ten-item multiple-choice questionnaire. Table 5 shows the questionnaire created for this assessment as a non-previous short validated questionnaire (maximum of 10 questions) was sufficient to cover information about opinion, satisfaction and motivation.

**TABLE 5:** Pre-Covid-19 Pandemic Questionnaire

Item	Question	Factor evaluated
1	Which task have you chosen more often to complete with Alcody?	Opinion
2	What is your favorite task with Alcody?	Opinion
3	What is your favorite activity?	Opinion
4	What is your favorite recipe?	Opinion
5	Do you like the help offered by Alcody?	Satisfaction
6	Are you satisfied with getting rewards?	Satisfaction
7	How much are you satisfied with your interaction with Alcody?	Satisfaction
8	Do you like learning how to program using Alcody?	Satisfaction
9	Do you think that Alcody motivates you to program?	Motivation
10	Do you think that you will be able to program with the help of Alcody?	Opinion

During the time that the teacher could be in class with the students, from September 2019 to March 2020, direct observation could be employed as well as a log of the programs coded by the students to find out how they were programming to recognize possible common mistakes and how they could overcome those mistakes.

From March 2020 to May 2020, it was not possible to have direct observation, given the COVID-19 pandemic. As students had been able to continue using Alcody, a shorter online anonymous questionnaire (five multiple-choice items) was created to find out their opinion, satisfaction and motivation as shown in Table 6, the registration logs of their activity when they connected online with their teacher and the programs created were also analyzed.

**TABLE 6:** Questionnaire During Covid-19 Pandemic

Item	Question	Factor evaluated
1	Do you think that you can concentrate at home using Alcody?	Opinion
2	Are you motivated to continue using Alcody?	Motivation
3	Do you like the help offered by Alcody?	Satisfaction
4	Do you like that Alcody helps you to deal with the COVID-19 pandemic?	Satisfaction
5	What recommendations do you want Alcody COVID to give you to help you continue to learn to program with it?	Opinion

Table 7 sums up the data collection techniques used for this research and the research question to be answered with the data analyzed.

**TABLE 7:** Data Collection Techniques

Period	Technique	RQ
Sept 2019 – Mar 2020	Pre-test & Post-test 1 pre-covid questionnaire direct observation students’ programs	1 2 2 2
mar 2020 – may 2020	second post-test questionnaire during covid alcody’s log students’ programs	1 2 2 2

### H. Analysis Procedure

To answer RQ1 and test Ha, a one-way ANOVA was used to analyze the difference in the means of the test scores in the three different months in which it was done, i.e, September, February and June:

\begin{equation*} \mathrm {y}_{\mathrm {ij}}=\mathrm {\mu +}\mathrm {\alpha }_{\mathrm {i}}+\varepsilon _{\mathrm {ij}}\end{equation*}

where $\mathrm {y}_{\mathrm {ij}}$ are observations for the dependent variable (with $y =$ {NotePre, NotePost1, NotePost2}), computed as the test scores; $\mu $ is the model mean; $\mathrm {\alpha }_{\mathrm {i}}$ is the main effect of level $i$ (the independent variable introduced in Section V.E., where each level depending on the use of the learning companion until time $i$) and $\varepsilon _{\mathrm {ij}}$ is the random error (where $i =$ September, February, June and $j$ the index related to the students).

In the case of the Conditions concept, the hypotheses were solved with a t-test for paired samples.

To answer RQ2, the answers to the questionnaires were analyzed together with the analysis of the programs, direct observations and registration logs.

### I. Validity and Reliability

Statistical calculations were performed with the program IBM SPSS Statistics Version 25. To test inter-rater reliability during the coding of the assignments using the rubric, Cohen’s kappa was used, see [115]. This statistic is one of the most commonly used to measure concordance between raters. Its value can range from −1 to 1. In the questionnaires examined, all items had Cohen’s kappa values over 0.80

In the questionnaire, the content validity in this study was determined by quantitative analysis of nine expert judgments, giving an Aiken V value greater than 0.7 in all items, which is considered acceptable, see [116]. A construct validity in pre-posttest was also carried out using exploratory factor analysis employing varimax rotation and the criterion of extracting eigenvalues >1. Cronbach’s alpha was also calculated, and its value was acceptable: 0.812 [114].

To strengthen the validity and reliability of the direct observation, two independent observers were taking notes during the face-to-face teaching sessions with Alcody (around 30 children per group, several sessions per week in their school’s timetable). The observers noted how engaged and motivated they perceived the children to be. Cohen’s kappa was calculated, its value being good: 0.625 [115].

## VI. Results

### A. Learning Gains

Any possible improvement in the scores of each individual between the pre-test, the first post-test before the COVID-19 pandemic, and the second post-test during the COVID-19 pandemic was studied, and, where necessary, quantified.

Given the different number of questions in tests as explained in Section IV.B (see Figure 10) two analyses were run. The first compared the input and output scores to the answers of Q1, Q2, Q3). The second compared the condition scores to the answers of Q4.

**FIGURE 11.**
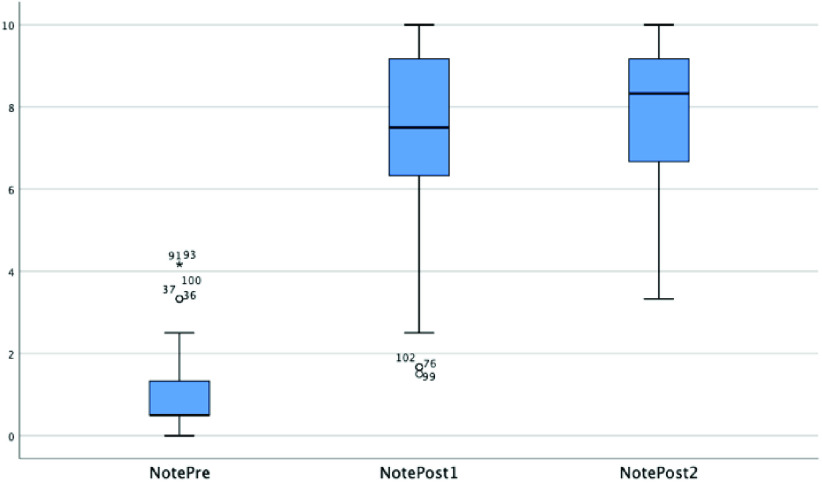
Pre- and post-test box plots for all concepts.

Figure 11 shows box plots in the pre- and two post-tests for the input/output questions (Q1, Q2, Q3). Small circles with a number represent atypical data. Table 8 shows mean and median values (more representative than mean in asymmetric distributions, as in this case) and standard deviations. The pre-test median value was 0.50, with a data dispersion of 0.85. The first post-test showed a greater data dispersion (2.05) but, in general, it showed much better results, with the median at 7.50. In the second post-test, the median increased to 8.33 but, with a data dispersion of 1.47, the scores are more concentrated. The results of the ANOVA are shown in Table 9.

**TABLE 8:** Descriptive Analysis of the Sample

TEST	N	$\bar {x}$	Med	SD
Pre	137	0.88	0.50	0.85
FIRST Post (PRE-COVID)	137	7.56	7.50	2.05
SECOND POST (DURING COVID)	137	8.01	8.33	1.47

**TABLE 9:** One-Way ANOVA

	Sum of squares	df	Mean squares	F	Sig.
Between groups	4368.43	2	2184.22	917.01	0.000
Within groups	971.81	408	2.39		
Total	5340.24	410

As evidenced, there were significant differences (p = 0.000) in the scores between the different levels for the input/output concepts. A post-hoc analysis was used to yield more information about these differences. Tukey’s HSD tests compared each score for the test at a moment of time (September, February, June) with every other score twice and showed significant differences (p < 0.05) between all pairs. The differences found between the test scores in the different months are all therefore statistically significant.

**FIGURE 12.**
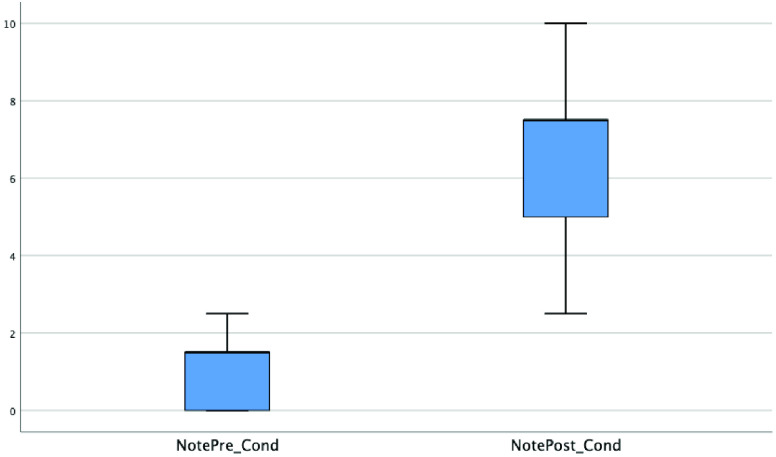
Box plots in pre- and post-test for the condition concept only taught online during the lockdown.

For the second analysis, to compare the scores for the condition concept, a paired t-test was carried out between the pre-test scores (September 2019) and the second post-test scores (June 2020) to see if there was a significant improvement in these scores too, taking into account that conditions is a concept that had only been explained online during the lockdown. Figure 12 shows box plots of the scores. Table 10 shows a descriptive analysis for these data.

**TABLE 10:** Descriptive Analysis of the Sample for the Condition Concept (Taught During the Lockdown)

TEST	N	$\bar {x}$	Med	sd
Pre	137	0.98	1.50	0.90
POST	137	6.97	7.5	1.92

Table 11 demonstrates the significant increase in scores even with this concept only having been taught during the lockdown, with p < 0.001. This increase was quantified using Cohen’s d. There is a huge effect (d = 4.01) with an 86% change percentage.

**TABLE 11:** Results of the Paired T-Test Between Pre-Test and Post-Test in the Condition Concept

t-test	df	p-value
30.093	136	< 0.001

#### 1) Opinions and Usage Patterns

As stated in Table 5, five items (1-4 & 10) of the questionnaire were focused on gathering information about students’ opinions. Three items (5-8) were focused on gathering information about students’ satisfaction. One item (9) was focused on information about students’ motivation. Table 12 sums up the results.

**TABLE 12:** Analysis of the Pre-Covid Questionnaire Results

Item	Question	Answer
1	Which task have you chosen more often to complete with Alcody?	Activities (49.6%), Recipes (26.3%), Questionnaires (24.1%)
2	What is your favorite task with Alcody?	Activities (59.1%), Recipes (36.5%), Questionnaires (4.4%)
3	What is your favorite activity?	Wolf, goat and cabbage riddle (50.4%), Missionaries and cannibals (19.7%), Jumping frogs (29.9%)
4	What is your favorite recipe?	Breakfast (35%), Omelet (11.7%), Hamburger (43.1%), Pasta (10.2%)
5	Do you like the help offered by Alcody?	Yes (93.4%)
6	Are you satisfied with getting rewards?	Yes (95.6%)
7	Are you satisfied with your interaction with Alcody?	Yes (98.5%)
8	Do you like learning how to program using Alcody?	Yes (93.4%)
9	Do you think that Alcody motivates you to program?	Yes (94.4%)
10	Do you think that you will be able to program with the help of Alcody?	Yes (92.7%)

Regarding opinion, 49.68% of students answered that they chose Alcody’s activities more than the recipes (26.28%) or chatting with Alcody (24.09%). When students were asked for their favorite Alcody activity, 50.36% answered the riddle of the wolf, goat and cabbage; 29.94% jumping frogs; and, 19.71% missionaries and cannibals.

When students are asked to choose their favorite recipe, 43.07% said the hamburger; 35.04% the breakfast; 11.68% the omelet; and, 10.22% the pasta.

Alcody’s help was found very useful by 71.53% of students; 21.9% answered that the help was useful. All the other students bar two were indifferent. Only two students said that the help given by Alcody was not useful at all because they had not needed it.

In regard to using Alcody to program, 64.23% of students thought that they were able to program with the help of Alcody, 23.4% thought that they would be able to program with the help of Alcody shortly, 7.3% stated that they were unsure if they would be able to program even with the help of Alcody, and 5.1% thought they would be able to program with the help of Alcody in the future.

Regarding satisfaction, students were highly satisfied with their interaction with Alcody and with the reception of rewards. Almost all (98.5%) students answered that they were satisfied with Alcody, and 95.6% of the students enjoyed having rewards, 93.4% enjoyed learning how to program using Alcody, and 94.4% considered that Alcody motivated them to learn how to program even during the COVID-19 pandemic when they were at home using Alcody online.

In Ecuador, the lockdown started in March 2020. Students were at home without the possibility of going out since then but could work with Alcody online during that time (one hour per week). They could progress with the condition concept and review the previous concepts with the chat and continue interacting with the recipes and activities. At the end of May 2020, students were asked to complete the questionnaire shown in Table 6; the results are shown in Table 13.

**TABLE 13:** Analysis of the Questionnaire Results During Covid

Item	Question	Most selected answer/ %
1	Do you think that you can concentrate at home using Alcody?	Yes (92%)
2	Are you motivated to continue using Alcody?	Yes (100%) – 7.84 mean value (0 min-10 max scale)
3	Do you like the help offered by Alcody?	Yes (98.5%)
4	Do you like that Alcody helps you to deal with the COVID-19 pandemic?	Yes (91.2%)
5	What recommendations do you want Alcody COVID to give you to help you continue to learn to program with it?	How to prevent infection by COVID (83.2%)

Students who answered that they think that they could concentrate at home was 92%. This could be the reason that explains that they were motivated to keep learning with Alcody. On a scale from 0 (minimum motivation) to 10 (maximum motivation) the average mean value was 7.84. This motivation level was even greater than before the lockdown as can be seen in Table 14.

**TABLE 14:** Comparison of Results Before and During Covid-19

Factor	Before	During
Motivation	94.4% (Q9)	100% (Q2)
Help	93.4% (Q5)	98.5% (Q3)
Opinion	98.5% (Q7)	92% (Q1)
Satisfaction	95% (Q5-Q8)	95% (Q3-Q4)

Regarding the help provided by Alcody, 98.5% of students liked the help during the COVID-19 pandemic: 56.9% to study inputs/outputs and the remainder to study conditions. The recommendations and help provided by Alcody in relation to the COVID-19 pandemic were appreciated by 91.2% of students.

They valued receiving information about how to prevent being infected by COVID-19 (83.2%), how to play during a health crisis (47.5%), tips on staying at home (58.3%), how to deal with parents working from home (34.3%), how to do the activities at home (50.3%), to acknowledge health professionals (32.1%) and how to learn online (49%).

During the direct observation with Alcody, the observers agreed that students were engaged and motivated during all sessions with Alcody. Both observers saw that students sat in front of their computers and they were completing the activities, following the recipes and writing the programs.

Sometimes when they had questions, students asked the teacher but most of the time they used the help provided by Alcody in the tutorials and worked hard for their reward. When they got a reward, they became really happy and excited.

Some correct programs written by students were: 

1 begin

2 write ”hello”

3 end

1 begin

2 write ”Type the value of a side:”

3 read A

4 B = A + A + A + A

5 write ”The perimeter is”,B

6 end

Some incorrect programs written by students were the following. They have been selected because they represent a common mistake made by at least ten students.

1 begin

2 write ”type the hello variable”

3 read hello

4 end algorithm

In this case, the mistake is related to the use of variables and p-code keywords. Students had, at the beginning, difficulties understanding the concept of a variable. It could be because it was a different use from Scratch. With Alcody, students had to write the instruction instead of dragging and dropping the blocks so they had to identify where to put the variable without the help of gaps in the instructions.

The second common mistake was to change the name of a p-code instruction. Given that in Scratch they did not have to type the instruction, they did not need to learn the word for it. With Alcody, many students asked if “end” and “end algorithm” were the same.

After six months of teaching most were finally able (as revealed by the post-test scores) to understand how to correctly use variables in the programs and p-code instructions. Some comments included:

• “Alcody is very useful for us. It is very interesting. It has good activities.”
• “It’s interesting because you can talk to Alcody and it’s not just a boring program.”
• “It’s important that Alcody reacts to feelings because people have a heart. They don’t have a heart of stone, and they have feelings.”
• “Alcody can get happy, bored or angry.”
• “I like the run button of Alcody and the debugging possibilities. If Alcody didn’t have them, I wouldn’t know what to do.”
• “Alcody is a way to improve my programming.”
• “Alcody helps us improve our learning with activities.”

It is important to highlight that students noticed and really appreciated the possibility of executing the programs with debugging and help, and the emotion management so that they did not feel that they were alone when writing programs, but acknowledge the emotional support of Alcody.

Students enjoyed the fun activities and following the recipes and whenever they completed some programs, they would ask for some time with the activities and recipes. Sometimes they felt frustrated when their program did not run as they expected, but Alcody’s emotional support and the debugging possibilities guided them toward completing the activity correctly. It seems that the reward given and the possibility of subsequent activities and recipes motivated them to keep trying more programs and thinking how to write the programs correctly to achieve the goals given by their ‘friend’, Alcody.

In March 2020, when the condition concept was about to be taught, classes had to stop. The COVID-19 pandemic forced everyone to remain at home. For some weeks, all teaching was cancelled. During that time, lessons were being prepared to be taught online, and teachers agreed to keep using Alcody online too.

Students were happy to keep using Alcody at home. The logs reveal that students connected online with their teacher and used Alcody at the same time as they would have done in class. When evaluating the programs written by the students, the average correct score was 7.77 on a scale from 0 (incorrect) to 10 (correct). Some correct programs written by the students were:

1 begin

2 write ”Type your age”

3 read age

4 if age >= 10

write ”You are older than 10”

else

write ”you are not older than 10”

5 end

Common mistakes are related to confused instructions such as the sample below in which the student has confused read with write but the general idea, use of variables and structure of the condition with input and output are correct.

1 begin

2 read ”Write your age”, age

3 if age >= 18 then

write ”You are an adult”

else

write ”You are not an adult”

4 end

Some comments provided by the students include:

• “I like Alcody to give advice about prevention and care.”
• “I like that Alcody explains to me why I should be at home.”
• “I like playing outside but having Alcody I can play with him and learn how to program.”
• “I like to be at home with my family and I can keep using Alcody online.”
• “I am sad because my grandmother is ill, but Alcody seems to understand that I am sad and it gives me some comfort so I keep using it.”

To sum up, before COVID-19 they used it in class with their classmates and teachers. They were focused on their task, engaged, motivated and happy to have the possibility to keep learning programming with a learning companion like Alcody.

During COVID-19, students used Alcody at home connected at the same time as their lessons (one hour per week) with their teacher. Students were able to create programs as before but make significant progress with new concepts such as conditions, while reviewing the previous concepts to create more complex programs with Alcody’s help.

It is important to highlight that both before and during the lockdown, the teacher only took on the role of an assistant in case students were unable to solve some issue with Alcody, but most of the time students were directly interacting with Alcody in class or at home using Alcody as their guide.

## VII. Discussion

### A. Evaluation of Results and Implications

Adaptability is an important element for computer-based learning environments in general, and involves the personalization of the learning process and the provision of feedback according to the needs of the learner. The question is how to design such computer-based learning environments [100]. How can a constructionist environment provide appropriate feedback to the learner without being strictly guided?

This paper proposes an answer to that question: with a learning companion based on Papert’s constructionism. The learning companion, in this case Alcody, is personalized for the students. It provides feedback according to the needs of each child and guides them toward the construction of their solution without telling the solution or providing a correct answer.

In this context, constructionism has a lot to offer both as a pedagogy that underpins the activities and as a theory of design that guides the development of the environment [24].

With Alcody, the possibility of interacting in natural language (p-code in this case) to write the programs, execute and debug them empowers children with the tools to reach their solution [117], without feeling alone, even in pandemic situations (they have Alcody on-line) and taking their emotions into account (with the emotional support of Alcody).

The results in Section VI.A answered **RQ1: Did students (aged between 10 and 12) increase their learning scores in a pre-post test using Alcody before and during COVID-19 pandemic?** For the input/output concept (Q1, Q2, Q3), participants improved significantly from the pre-test to the first post-test (before COVID-19) and the second post-test (during COVID-19). Additionally, scores in the second post-test showed a significant improvement over the first post-test. For the condition concept (Q4), participants also improved significantly from the pre-test to the second post-test (during COVID-19) with an 86% change percentage.

The reason why conditions were so well learned could be that the condition concept is highly related to the real-life condition concept and children during the lockdown had a lot of resources at home to interact with. For instance, if the condition instruction was, “if I drink all water in the glass, the glass will be empty.” Children could drink all water from the glass of water to see an empty glass. This is grounded in the principles of Ausubel’s Meaningful Learning Theory [118].

Moreover, the fact that children were at home with all their physical resources and objects, and concentrated on just the interaction with the program (no distractions from other classmates talking or making jokes) made the teaching of conditions easier as recorded by the observers, even during a complex lockdown.

The results in Section VI.B answered **RQ2: How satisfied were students interacting with Alcody before and during the COVID-19 pandemic? What was their opinion of Alcody? Were they motivated to use Alcody even during the lockdown?** Overall, they were satisfied as 95% of the students were motivated to use Alcody before COVID-19 (average calculated from questions 5 to 8, Table 12) and 95% remained motivated to continue learning with Alcody (average calculated from questions 3 to 4, Table 13). Students used Alcody before and during the COVID-19 pandemic and their motivation and opinion remained the same (over 90%) as shown in Table 14.

The results achieved in this paper are similar to the results found in [119] who provided positive results when, undergraduate students in their case, learned Java programming by using a learning companion as an Eclipse plug-in. They did not use an interactive learning companion with emotional support, but just the presence of the learning companion’s hints on the screen seemed to provide positive results in the teaching.

In their paper, they concluded that learning companions have the potential to become a dexterous tool in the learning process [119]. To reach that potential, nine guidelines can be followed [19]. The guidelines are discussed here, as their work is similar to us, based on constructionism to teach programming, but with the main difference that they used Scratch in their work and, this paper is focused on using a learning companion, after students had used Scratch, even where they could not attend to face-to-face lessons because of the COVID-19 lockdown.

The reformulation of the nine guidelines as recommendations and implications in the case of students who have already used Scratch and are interacting online with a learning companion are the following:

#### 1) Social Interaction

They said that collaboration between team members is a vital part of coding activities. Where this social interaction is not possible due to the lockdown, this paper proposes the use of a social learning companion that can continue teaching programming to children according to Papert’s constructionism.

#### 2) Appropriate Design According to Age

Different age groups (kids and teens) need different approaches and designs in order to engage with a coding activity. Alcody allows both the environment and the activities to be personalized. With the dialog, children are asked to construct their program and are guided toward their solution according not only to their age but also to their particular answers.

#### 3) Duration of the Activity

According to constructionism [12], when children use technological tools, duration is key for them to become personally, intellectually, and emotionally involved. Alcody was used online during the lessons in class following the same schedule as during the lockdown. The duration of the activity should be enough for them to reach a solution but not too long that they become bored. Students should be allowed to play according to the gamification rewards to keep motivated.

#### 4) Relevance of the Activity and Meaningful Content

Offering a supportive theme for the artifact creation process, in which participants can meaningfully participate in real-life settings, is a key factor in supporting the psychological and sociocultural elements for effective learning. The problems are asked to be solved by programming should be extracted from real-life situations. For instance, the learning companion could ask the students to write a program to calculate how much they will spend on presents for their friends based on the cost of each present and the number of friends.

#### 5) Physical and Digital Artifacts

It seems that the inclusion of physical tasks is engaging and enabled participants to enhance their skills. When using Alcody, some children used pen and paper to draw ideas to help them program and other students looked for physical objects their home to understand the real-life examples provided. This is recommended to help students understand the programming concepts.

#### 6) Children’s Attitudes and Motivation

The learning process should be supported by tasks that encourage children to reflect, motivate them to collaborate, and give them meaningful reasons to complete their artifacts. Alcody asks children to write programs that are meaningful for them and encourages them to reflect on their programming. No correct solution should be provided. On the other hand, children have to think to write their program and execute it to check whether it works. If the program works, they are motivated. If they feel sad, they can tell this to the learning companion that will give them a recommendation to feel better and try the program again with the help of the debugging and tutorials until they understand what they are doing wrong and change it to reach their own correct solution that works (following the two core ideas of learning and doing and getting things done with a positive attitude).

#### 7) Cognitive Overload

Coding activities for children can have a high cognitive load, which affects their performance and overall experience with the tasks. Alcody reduces the cognitive load by providing just one program at a time. Firstly, the programs only focus on one concept such as input/output or conditions. As children manage to solve those problems, more complex programs that combine several concepts will be offered. If the learning companion detects that a child is overloaded, sad, frustrated or bored, a comment should be provided to help them reach a better mental state to keep programming with sentences such as “some things are complex at beginning but when you practice, you will be able to solve any problem!” In the words of Papavlasopoulou *et al.* [26]: “Students should be persistent, put effort in, and deal with difficulties; therefore, having positive attitudes and keeping themselves motivated result in better management of their cognitive load.

#### 8) Appropriate Tasks

The tasks should make children both interested and able to learn. As indicated in point 4, programs in Alcody are related to meaningful situations for children so that they get motivated but here Alcody offers gamification as an additional reward to maintain motivation. Tasks should not only be related to programming as writing programs but also as games to think how to program. For this reason, the learning companion should have the possibility of following a recipe as a metaphor for a program [55] playing with the ingredients, a cooking task that is related to real-life, meaningful and suitable to think like a programmer. Furthermore, the learning companion should have games like riddles to keep children thinking logically, spontaneous tasks that maintain motivation, and give them rewards that are interesting to them.

#### 9) Meaningful Framework for the Involvement of the Instructors

In the construction of an artifact, children are not alone: practitioners (e.g., teachers and assistants) and anyone else who is responsible for the learning task are also involved. As noted by Papert [12], “sharing the problem and the experience of solving it allows a child to learn from an adult not ‘by doing what teacher says’ but ‘by doing what teacher does.”’ Before the lockdown, children were in their class talking about how to solve the problems presented by Alcody, following the tutorials and completing the spontaneous games. The teacher was available, only as a support to the children, if they had any problems. Equivalently, during a lockdown or whenever face-to-face interaction is not possible, children can connect online with their learning companion in a videoconference with their teacher as support to solve any problem and share their comments that avoids any feeling of isolation, and having even more support with their parents at home thanks to technology that serves to keep developing children [120].

### B. Threats to Validity

The study presented some threats to validity [121], [122]:

• **Construct validity**: Some novel effects may be present, since the use of a computer tool in learning can always be attractive and novel, something that is usually out of their routine. When an innovation is introduced, it can breed excitement and enthusiasm that contributes to success, especially if little innovation has occurred 123]. Also, the instrumentation is based on questionnaires to measure cognitive-emotional processes. These are complex phenomena and they are not easy to analyze. The use of physical or multi-modal methods could give additional information [124].
• **External validity**: Since the groups are not random, the study does not have certainty that the sample is representative of the general population. In this research, although students are not in a single scenario (data were collected when they were in class but also at home), it would not be appropriate to generalize this to all students of their age, taking into account, in addition, the exceptional situation of global pandemic that arose during the development of the experiment. Alcody should be tested with children without previous programming knowledge, and with a control group. In single pre-post test research design without a control group, the degree of control is low [125]. With a control group, the degree of control would be higher. However, sometimes with human participants it is not possible to have such a high degree of control [126].
• **Internal validity**: Although in this pre-post test single group, some of the internal validity are ruled out automatically, for example differential rates of mortality and selection bias, see [127], [128], some maturation effects may be presented, such as getting older or more experience. Even in short studies such processes are a problem [122]. Another threat is the digital skills involving students, especially at the time of lockdown, where the teacher is present only remotely and the computer equipment available at home may not be the same for each student. Although all children reported that they had a computer, some could be faster than others. Similarly, the Internet connection could be faster in some homes.
• Conclusions validity: Cook and Campbell [121] called it statistical conclusion validity (SCV). They stressed that SCV “is concerned with sources of random error and with the appropriate use of statistics and statistical tests”, so there is no perfect validity. Although a descriptive analysis is provided together with all assumptions needed in the inferential studies, type 1 errors (incorrect rejection of null hypothesis) and type 2 errors (the failure to reject a false null hypothesis) are a part of statistical tests, a perfect result cannot be ensured, although it can be minimized. Reliability tests of the questionnaire’s design and the pre-post test process have been presented. Assumptions about normality (required for all parametric test used) and homoscedasticity (also required in the ANOVA procedure) in the data have also been tested. Despite this, some threats to conclusion validity can be found in the lack of a control group in the experiment. A restriction of range could also be another threat to SCV given the use of a rubric with a finite and discrete number of values for the scoring of the tests (see Table 4).

## VIII. Conclusion and Future Work

In an experiment with 137 students aged between 10 and 12 using the learning companion Alcody to continue learning programming for ten months (even during the COVID-19 pandemic), students were able to significantly increase their learning scores in a pre-post test on input/output programs from no knowledge in the pre-test with a 0.88 average score, up to a 7.56 average score in the first post-test in February before the lockdown and up to 8.01 in the second post-test in June during the lockdown.

From the analysis of the pre-test and post-test scores during the lockdown of the condition concept that was only explained online, a significant increase in average score from 0.98 to 6.97 was recorded.

Regarding the satisfaction levels, it can be concluded that students were highly satisfied using Alcody as 95% of the students were satisfied before and during COVID-19. From direct observation in the class (from September 2019 to March 2020), it can be concluded that students were focused on their task, engaged, motivated and happy to have the option to continue learning programming with a companion like Alcody. They rarely needed to ask their teacher about something they did not understand; they were able to solve their issues with Alcody.

From March 2020 to May 2020, students continued to use Alcody online at home and solved problems, even with the increased difficulty of combining input and output instructions with conditions. Again, the teacher was connected online as an assistant for any problem or issue that could not be solved by Alcody, but students rarely asked the teacher as they had the tutorials and the help provided by Alcody.

The role of the teacher was focused on creating the questions for students to answer in Alcody and observing how students wrote the programs to find out common mistakes in order to provide more questions on programs focused on those concepts.

A main limitation of this study has been the impossibility of having a control group that did not use Alcody. It is our intention that, when the COVID-19 situation passes, to contact other schools to have a control group, and to test if the results are the same with more concepts such as loops and combinations of concepts.

Another line of future work is to carry out a study to investigate the relationship between the improvement of the learning scores and the satisfaction levels of the students, even when they do not have any previous Scratch or programming experience.
